# The influence of fracture severity on postoperative outcome and quality of life after locking plate fixation of proximal humeral fractures

**DOI:** 10.3205/iprs000164

**Published:** 2022-05-23

**Authors:** Simon Thelen, Jan P. Grassmann, Madeleine Schneider, Carina Jaekel, Dana M. Meier, Marcel Betsch, Mohssen Hakimi, Michael Wild

**Affiliations:** 1 Department of Orthopedics and Trauma Surgery, Medical Faculty, Heinrich-Heine-University Düsseldorf, Düsseldorf, Germany; 2 Department of Orthopedics, Trauma and Hand Surgery, Klinikum Darmstadt, Darmstadt, Germany; 3 Department of Orthopedics and Trauma Surgery, University Hospital Mannheim, Mannheim, Germany; 4 Vivantes Klinikum Am Urban, Department of Orthopedic, Trauma and Hand Surgery, Berlin, Germany

**Keywords:** proximal humeral fracture, fixed-angle plate osteosynthesis, humeral head necrosis, outcome, quality of life, complications, fracture classification

## Abstract

**Objective:**

For proximal humeral fractures open reduction und internal fixation (ORIF) with a fixed-angle plate is considered the gold standard for surgical management. However, it can lead to poor functional outcomes and is associated with postoperative complications. Therefore, the purpose of this study was to investigate the influence of fracture severity by applying a new classification (simple versus complex) on clinical outcome and quality of life after ORIF of proximal humerus fractures.

**Methods:**

We conducted a prospective clinical study with an average follow-up period of 12 (SD 1) months after ORIF of proximal humeral fractures with a fixed-angle plate. The postoperative function and quality of life was measured using the Oxford Shoulder Score (OSS) and the Constant Score. Data was tested for statistical significance with the Mann-Whitney test and Fisher's exact test. Based on the findings of this study a simplified fracture classification system has been developed.

**Results:**

Seventy-two patients with a mean age of 65 years (SD 12) with 69% being males were included. According to the Neer classification, 35% (n=25) non-displaced (“one-part fractures”), 19% (n=14) two-part fractures, 15% (n=11) three-part fractures and 31% (n=22) four-part fractures were detected. Regarding the AO/OTA classification, 18% (n=13) were type A fractures, 43% (n=31) type B and 39% (n=28) type C fractures. From these criteria we derived our own fracture classification, including 50% (n=36) simple and 50% (n=36) severe fractures. Patients with simple fracture types achieved significantly higher total values in the Constant Score as well as the OSS (p=0.008; p=0.013). The cumulative incidence of complications in the entire patient collective was 14% (n=10) with humeral head necrosis (n=5) occurring only in the severe fracture group.

**Conclusions:**

The postoperative clinical outcome as well as the incidence of humeral head necrosis after ORIF of proximal humeral fractures with a fixed-angle plate correlates with the fracture type and severity. The newly derived fracture classification into simple and severe fractures is suitable with regard to clinical results and complication rate. However, prospective studies comparing ORIF vs. conservative treatment of proximal humeral fractures of the same severity are required.

**Level of Evidence:**

III

## Zusammenfassung

**Einleitung:**

In der chirurgischen Behandlung proximaler Humerusfrakturen gilt die offene Reposition und interne Fixation (ORIF) mit einer winkelstabilen Platte als Goldstandard. Allerdings kann das operative Vorgehen auch mit schlechten funktionellen Ergebnissen und postoperativen Komplikationen verbunden sein. Ziel dieser Studie war es daher, den Einfluss der Frakturschwere auf das klinische Ergebnis und die Lebensqualität nach ORIF von proximalen Humerusfrakturen anhand einer vereinfachten, eigenen Klassifikation (Einteilung nur in „einfach“ versus „komplex“) zu untersuchen.

**Material und Methoden:**

In einer prospektiven klinischen Studie wurden Patienten mit einer proximalen Humerusfraktur und Versorgung mittels einer winkelstabilen Plattenosteosynthese mit einer durchschnittlichen Nachbeobachtungszeit von 12 Monaten (SD 1) eingeschlossen. Die postoperative Funktion und Lebensqualität wurden anhand des Oxford-Shoulder-Score (OSS) und des Constant-Score gemessen. Die Daten wurden mit dem Mann-Whitney-Test und dem exakten Fisher-Test auf statistische Signifikanz überprüft. Basierend auf den Erkenntnissen dieser Studie wurde ein vereinfachtes Frakturklassifikationssystem entwickelt.

**Ergebnisse:**

Es konnten 72 Patienten mit einem Durchschnittsalter von 65 Jahren (SD 12), davon 69% Männer, eingeschlossen werden. Nach der Neer-Klassifikation waren 35% (n=25) der Frakturen nicht disloziert („einteilige Frakturen“), 19% waren (n=14) Zweifragmentfrakturen, 15% (n=11) Dreifragmentfrakturen und 31% (n=22) waren Vierfragmentfrakturen. Bezüglich der AO-Klassifikation waren 18% (n=13) Typ-A-Frakturen, 43% (n=31) Typ-B- und 39% (n=28) Typ-C-Frakturen. Aus diesen Kriterien haben die Autoren ihre eigene Frakturklassifikation abgeleitet, die 50% (n=36) „einfache“ und 50% (n=36) „schwere“ Frakturen umfasste. Patienten mit einem einfachen Frakturtyp erzielten sowohl im Constant-Score als auch im OSS signifikant bessere Gesamtwerte (p=0,008; p=0,013). Die kumulative Inzidenz von Komplikationen im gesamten Patientenkollektiv betrug 14% (n=10), wobei eine Humeruskopfnekrose (n=5) nur in der Gruppe mit schweren Frakturen auftrat.

**Schlussfolgerungen:**

Das postoperative klinische Ergebnis sowie die Inzidenz von Humeruskopfnekrosen nach ORIF von proximalen Humerusfrakturen mit winkelstabiler Platte korrelieren mit dem Frakturtyp und der Frakturschwere. Die neu abgeleitete Frakturklassifikation in einfache und schwere Frakturen scheint hinsichtlich der Prädiktion klinischer Ergebnisse und der Komplikationsrate ein geeignetes Mittel zu sein. Allerdings sind prospektive Studien erforderlich, welche die operative gegenüber der konservativen Behandlung von proximalen Humerusfrakturen des gleichen Schweregrades anhand dieser Klassifikation weiter vergleichen.

## Introduction

Proximal humeral fractures are among the most common fractures, with an incidence rate of around 5% and an exponential increase in elderly patients [1], [2], [3]. Due to demographic changes as well as the age-related occurrence of osteoporosis, the incidence of proximal humeral fractures will further increase in the future [1], [4]. Treatment strategies for proximal humeral fracture are commonly based on the Neer classification, which is centered around the four anatomical segments of the proximal humerus and the extent of fracture dislocation [5], [6], [7], [8]. However, clinical evidence concerning surgical or conservative management of proximal humeral fractures is generally very sparse [9], [10]. Simple fractures with no or minimal displacement are often treated conservatively, while displaced fractures are predominantly treated operatively with either fixed-angle plate fixation, intramedullary nail osteosynthesis or joint replacement [11]. Open reduction and internal fixation (ORIF) with fixed-angle plates is currently considered the gold standard in the treatment of most types of proximal humeral fractures, especially displaced two-, three- and four-part fractures [12], [13]. Over the past decades, surgical equipment, implants and techniques have improved significantly. Fixed-angle plates offer enhanced stability due to the locked screw-plate interface and polyaxiallly angulated fixed-angle screws, which improve fixation and pullout strength in osteoporotic bone [14]. Also, large and solid tuberosity fracture fragments can be treated surgically with plate osteosynthesis [15]. However, poor bone quality can make screw implantation technically challenging and failure of the osteosynthesis with subsequent screw cut-out and/or humeral head necrosis may occur. Recent studies documented fairly high rates of surgery-related complications including secondary fracture displacement, screw cut-out, intraarticular screw migration and avascular humeral head necrosis after operative treatment of proximal humeral fractures [16], [17], [18]. A systematic review in 2011 demonstrated a rate of avascular head necrosis of 11% after fixed-angle plate osteosynthesis [19]. The higher complication rates in cases that were treated with fixed-angle plate fixation could be explained by the severity of the fracture itself and not solely by the treatment method applied. Previous studies suggest that fixed-angle plate osteosynthesis, even in more severe fracture types, demonstrated results comparable to those of conservative therapy, which is mainly used for simple fracture types [19], [20], [21], [22]. The poor results after fixed-angle plate osteosynthesis in many studies may show a certain underlying bias, because most studies included only severe fracture types, while simpler fractures were treated conservatively. The fracture severity should be considered when interpreting these studies in order to compare “apples to apples”. Therefore it should be discussed if simple fracture types could benefit from surgical treatment in direct comparison to conservative therapy. Previous studies investigating the relationship between fracture severity and clinical/functional outcome after fixed-angle plate fixation are lacking. Hence, the aim of this study was to compare the clinical and functional outcomes, quality of life and complication rates after fixed-angle plate osteosynthesis of simple fracture types with those of severe proximal humerus fracture types. The authors hypothesize that fracture severity influences the outcome in patients with ORIF of proximal humeral fractures. 

## Materials and methods

**Figure 1:**
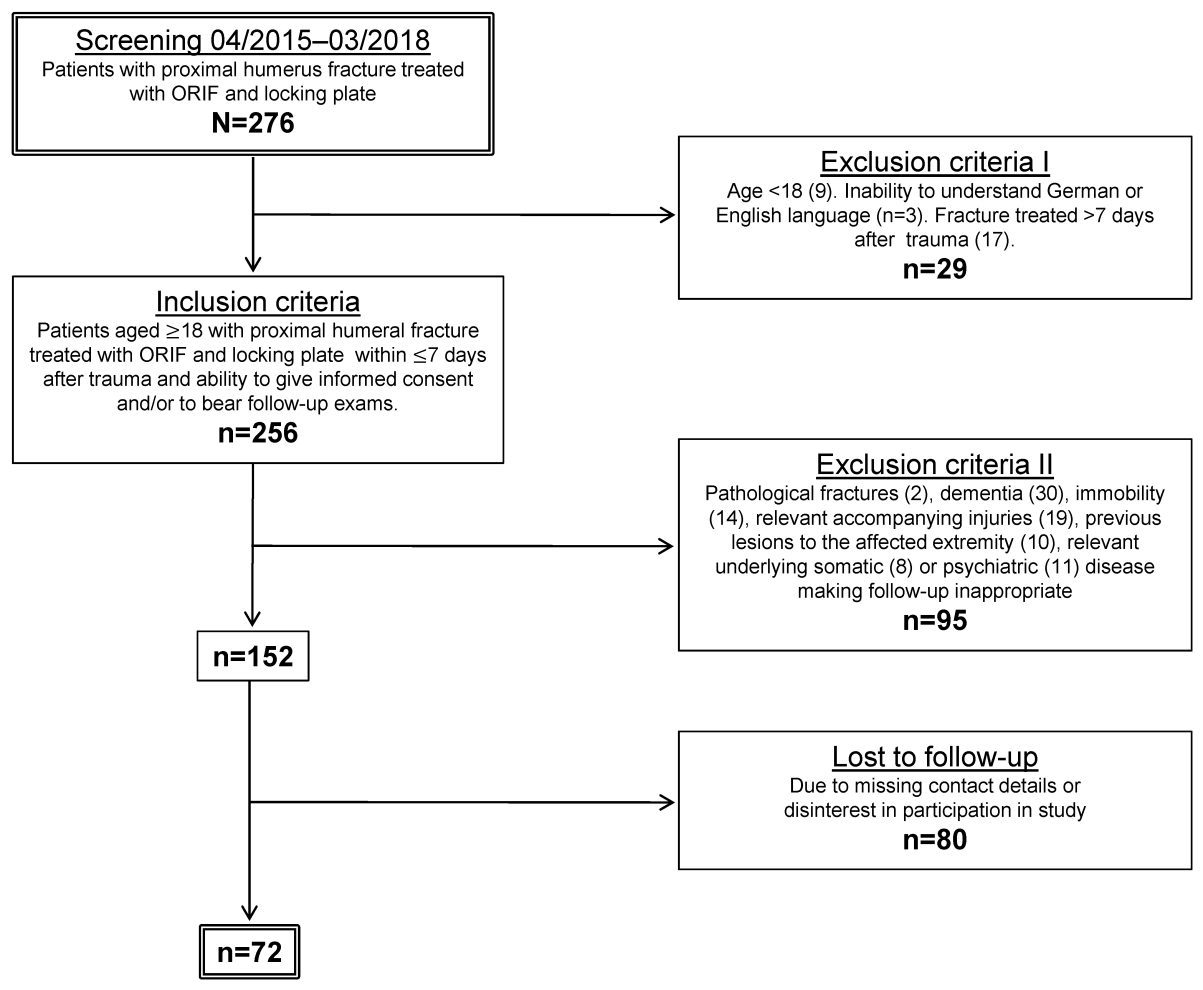
Figure 1: Flowchart of in- and exclusion criteria Initially, 276 patients were screened for having their proximal humeral fracture treated with ORIF and locking plate in a prospective 36-months period. 124 patients (45%) had to be excluded for various reasons that made participation inappropriate or posed a risk of bias due to accompanying injuries or diseases. Another 80 patients were lost to follow-up, leaving 72 study participants.

We conducted a prospective single-center clinical trial and the study protocol was approved (study number: 5623) by the local institutional review board (IRB). From April 2015 to March 2018, 276 patients had undergone ORIF of a proximal humerus fracture with a fixed-angle plate within 7 days of the injury. Inclusion criteria for the patients were a minimum age of 18 years, signed written consent as well as surgical treatment of a proximal humeral fracture with ORIF. Exclusion criteria were pathological fractures, concomitant injuries or previous injuries to the affected extremity, as well as underlying diseases with significant functional impairment such as rheumatoid arthritis or immobility. See Figure 1 (Fig. 1) for details on inclusion and exclusion criteria. The mean follow-up was 12 months (SD 1) postoperatively.

### Baseline data

Patient age, gender, handedness, affected side and postoperative complications were recorded.

### Fracture classification 

Fractures were classified by applying the classification system of Neer (four-segments-classification) and the AO/OTA classification (A, B and C classification) [7], [23]. For further evaluation and in order to achieve a simpler, more intuitive and comprehensive classification, one- and two-part fractures (according to Neer) as well as type A and type B fractures (according to AO/OTA) were grouped as *simple fracture types*. Three- and four-part fractures (Neer) as well as type C fractures (AO/OTA) were grouped as *severe fracture types*. Furthermore, the fractures were divided into two groups concerning vascularity and stability-related aspects based on the observations of Hertel et al. [24]. The presence of one of the following criteria was sufficient for classification as a severe fracture type:

1. a continuous fracture line through the *collum anatomicum*,
2. a varus angulation of the fracture of > 20° and
3. a glenohumeral dislocation.

### Clinical outcomes

The Constant Score, the normalized Constant Score and the Oxford Shoulder Score (OSS) were recorded in all patients [25], [26], [27]. The objective range of motion (ROM) of the affected shoulder was measured with a goniometer (Burg-Wächter^®^ Model TARA PS 7600). The shoulder strength was measured by positioning the patient’s arm in 90° abduction and 30° anteversion with pronated forearm and a dynamometer fixed scale on the wrist. The patient was then asked to elevate his arm against resistance. The maximum weight (kg) which could be lifted and held up painlessly for at least three seconds was measured. ROM and weight values were then integrated into the functional scores.

### Statistical analysis

For statistical analysis, the software tools Microsoft^®^ Excel^®^ 2016 (Version 1907, Microsoft^®^, Redmond, Washington, USA) as well as IBM^®^ SPSS^®^ (Version 25, IBM Inc., Armonk NY, USA) were used. The Kolmogorov-Smirnov test was used to test for normal distribution of the data. Data were tested for statistical significance with Mann-Whitney test or Fisher’s exact test. A p-value ≤0.05 was considered significant. 

## Results

### Study population und baseline data

Of a total of 276 patients that were treated surgically, we were able to include 72 patients based on our inclusion and exclusion criteria. The main reasons for exclusion were dementia and other underlying somatic or psychiatric diseases that made informed consent or follow-up inappropriate, followed by relevant injuries or conditions affecting the ipsilateral extremity. Other patients were lost to follow-up due to missing contact details or disinterest in further participation (see Figure 1 (Fig. 1)). Out of the 72 individuals included, there were 69% (n=50) females and 31% (n=22) males. The mean age was 65 (SD 12) years. The dominant arm was affected in 47% (n=34). Concerning baseline data, there were no statistically significant differences between the groups (p>0.05).

### Fracture classification

**Figure 2:**
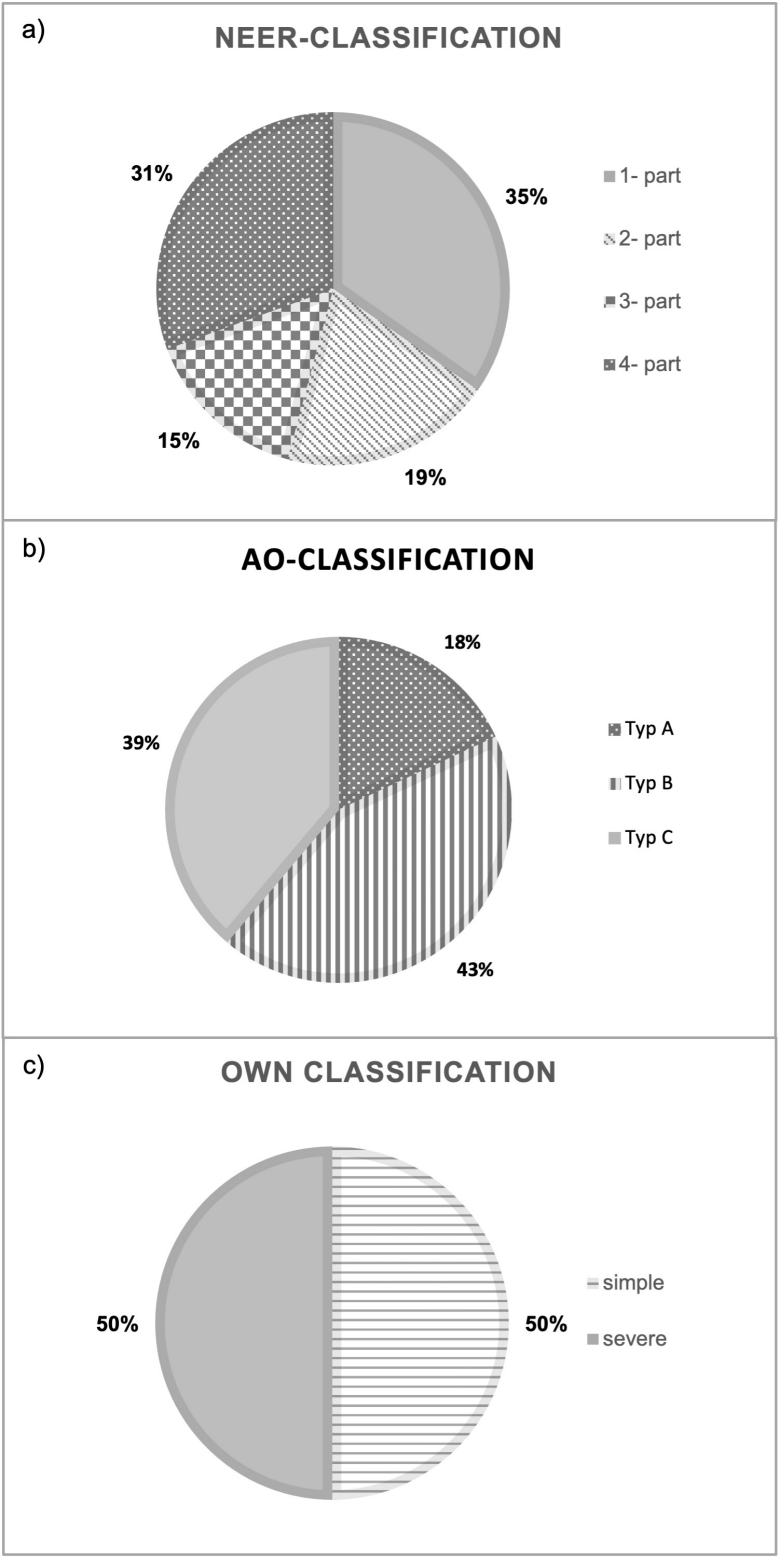
Fracture classification The proximal humeral fractures were classified differentiated according to the Neer Classification (a), the AO/OTA Classification (b) and the authors’ own simplified classification.

According to the Neer classification, 35% of the fractures were (n=25) one-part fractures (i.e. a fracture with less than 1 cm of displacement and less than 45° of angulation), 19% (n=14) two-part fractures, 15% (n=11) three-part fractures and 31% (n=22) were four-part fractures. This corresponds to a distribution of 54% (n=39) simple fractures and 46% (n=33) severe fractures (Figure 2a (Fig. 2)). Using the AO/OTA classification, 18% (n=13) fractures could be assigned to type A, 43% (n=31) to type B and 39% (n=28) to type C fractures. This results in a distribution of 61% (n=44) simple and 39% (n=28) severe proximal humerus fractures (Figure 2b (Fig. 2)). According to the criteria of our own classification, there were 50% (n=36) simple and 50% (n=36) severe fractures (Figure 2c (Fig. 2)).

### Clinical outcome

**Figure 3:**
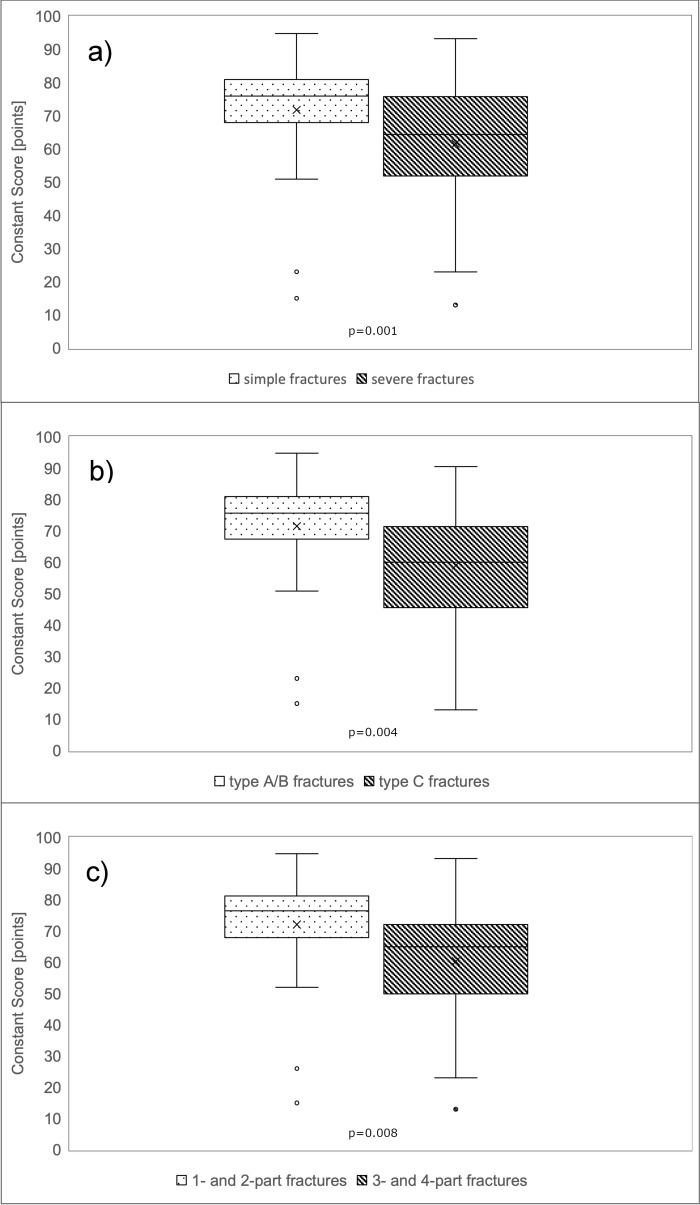
Figure 3: Functional results according to Constant Score Patients with simple fractures according to the authors’ own classification achieved significantly better results in all three different functional scores.

**Figure 4:**
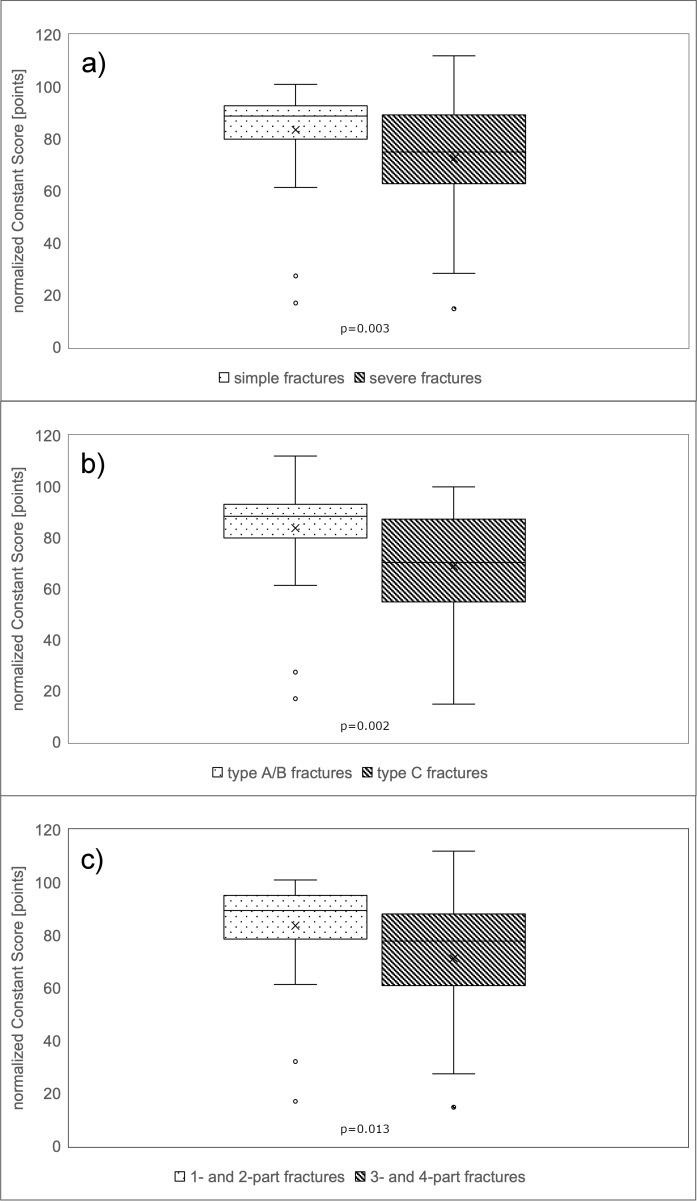
Functional results according to normalized Constant Score Patients with simple fractures according to the authors’ own classification achieved significantly better results in all three different functional scores.

**Figure 5:**
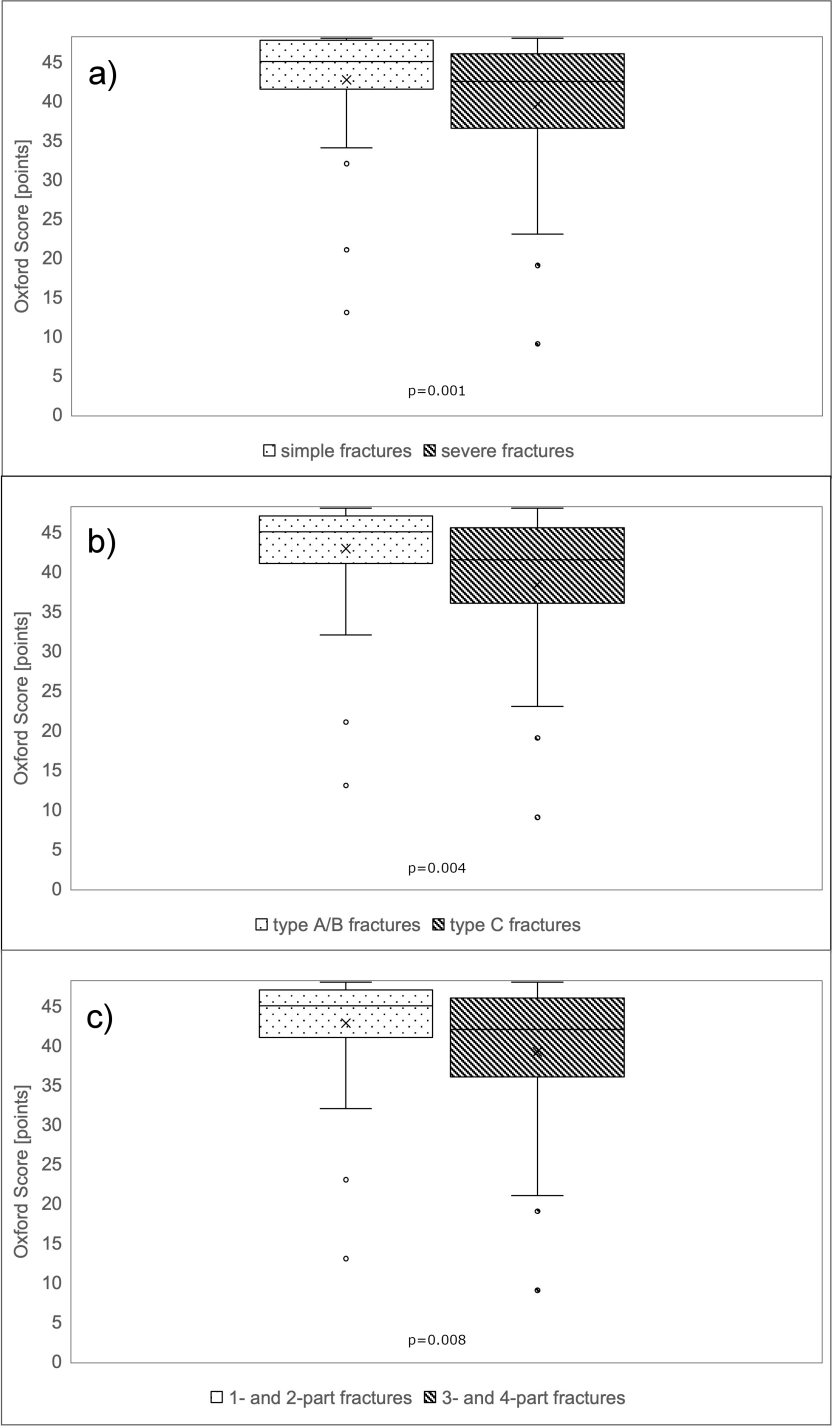
Functional results according to Oxford Shoulder Score Patients with simple fractures according to the authors’ own classification achieved significantly better results in all three different functional scores.

Regarding the Constant Score, patients averaged values of 67 (SD 18) points with a maximum score of 100 points. 6% (n=4) obtained very good (= 86 points) and 47% (n=34) good results (85–71 points). 25% (n=18) reached satisfactory (56-70 points) and 22% (n=16) poor results. The patient group with one- and two-part fractures according to the Neer classification achieved an average of 72 (SD 16) points in the Constant Score, whereas the patient group with three- and four-part fractures obtained significantly less points with an average of 60 (SD 19) points (Mann-Whitney U Test: p=0.001; Table 1 (Tab. 1), Figure 3a (Fig. 3)). A significant disadvantage of patients with a severe fracture type could also be recorded with regard to the AO/OTA classification (Mann Whitney U Test: p=0.004; Table 1 (Tab. 1), Figure 3b (Fig. 3)). Patients with type A and B fractures scored an average of 72 (SD 15) points. In contrast, patients with type C fractures achieved only 59 (SD 20) points. According to the authors’ newly defined classification, patients with simple fractures reached 72 (SD 16) points compared to patients with severe fracture types who achieved 62 (SD 19) points, also with a significant difference between both groups (p=0.008; Table 1 (Tab. 1), Figure 3c (Fig. 3)). Similar results were found for the normalized Constant Score, where patients with severe fracture patterns scored lower (p=0.013; Table 1 (Tab. 1); Figure 4a–c (Fig. 4)). Regarding the Oxford Shoulder Score (OSS), patients in general achieved an average of 41 (SD 8) points with a maximum score of 48 points. A total of 79% (n=57) of patients achieved good results (48–37 points), 14% (n=10) satisfactory (25–36 points) and 7% (n=5) poor results (≤24 points). In all of the fracture classification systems applied here, patients with simpler fracture types consistently reached significantly better results than patients with severe fracture types regarding all outcome scores (Table 1 (Tab. 1), Figure 5a–c (Fig. 5)).

### Complication rates

The cumulative incidence of postoperative complications following fixed-angle plate osteosynthesis in the entire patient collective was 14% (n=10). Overall, more complications occurred in patients with severe fracture types than in patients with simple fracture types, with no significant difference regarding the overall complication rate (p>0.05). In summary, the following complications could be observed: 7% (n=5) humeral head necrosis, 3% (n=2) arthrofibrosis, 1% (n=1) impingement-syndrome, 1% (n=1) secondary bleeding, 1% (n=1) impaired wound healing. As mentioned above, the incidence of postoperative humeral head necrosis, which accounted for 50% of all complications, was 7% in the entire patient collective and only occurred in patients with severe fracture types. Thus, significantly more patients were affected by humeral head necrosis in the group of severe fracture types than in the group of simple fracture types (Mann-Whitney U Test: Neer classification 0.017, AO/OTA classification 0.007, own classification 0.023). Cases of delayed union, malunion or nonunion were not observed in our cohort. 

## Discussion

The key findings of this study indicate that the postoperative clinical outcome as well as the incidence of humeral head necrosis after fixed-angle plate fixation of proximal humerus fractures correlate with the severity of the fracture. Furthermore, a proposal for an easily applicable classification system for the clinical differentiation into simple and severe proximal humeral fractures has been derived from this study population.

Epidemiological studies show that particularly elderly and female patients are affected by proximal humerus fractures [28], [29]. Thus, the group of patients included in the present study represents the typical age (65 years, SD 15) and gender distribution (69% female). 

In general, treatment strategies of proximal humerus fractures are classified as surgical and non-surgical/conservative. Evidence-based data or guidelines on how to treat which fracture type, either surgically or conservatively, are still missing [30]. Most non-displaced and minimally displaced proximal humeral fractures are treated with conservative methods, achieving good outcomes [31]. In contrast, displaced and fragmented (three- and four-fragment) fractures often require surgical intervention, including open reduction and plate fixation, minimally invasive plate osteosynthesis, intramedullary nail fixation, or shoulder joint arthroplasty [32]. Most current studies focus on the treatment of severe fracture types, because these fracture types may seriously impair the quality of life and lead to additional complications [33]. The few available randomized controlled trials (RCTs), as well as Systematic Reviews (SR), comparing conservative versus operative treatment of proximal humerus fractures focus mostly on complex fracture types, namely displaced three- and four-part fractures [33], [34], [35], [36], [37], [38]. In summary, all studies concluded: *“there is no difference in functional outcomes. Further high quality RCTs are required to determine if certain subgroup populations benefit from surgical management”* [33], [34], [35], [36], [37], [38]. The SR of Handoll et al. is to date the only SR which also considers non-displaced, simple fracture types [10]. However, those RCTs that include simple fractures usually examine different conservative follow-up schemes instead of comparing conservative and operative treatment strategies [39], [40], [41], [42]. 

The largest RCT to date on proximal humerus fractures, the ProFHER Trial showed no benefit to surgery versus conservative treatment overall for these injuries, including all fracture types [43]. Studies focusing on conservative treatment compared to fixed-angle plate osteosynthesis for only simple fracture types are still pending. This is definitely crucial, since the clinical outcome of a fixed-angle plate fixation depends on the severity of the fracture as shown in our study. Previous studies recommend conservative therapy for simple fractures of the proximal humerus – so what should be the advantages of a fixed-angle plate osteosynthesis for these fractures? Non-surgical treatment usually involves an immobilization period, followed by physical therapy and self-exercises. The immobilization of the shoulder in a sling provides support and pain relief during healing. However, there is a considerable risk of stiffness and long-lasting painful restriction of movement of the affected shoulder. Early mobilization is one of the concepts to avoid such undesirable results [39]. However, such early mobilization can also be associated with secondary fracture dislocation. Bockmann et al. identified age of more than 65 years as an important risk factor for secondary displacement in patients with fractures involving the larger tuberosity [44]. 

Various classification systems are routinely used in clinical practice in order to better describe the fracture morphology and, ultimately, to improve and standardize the treatment of proximal humeral fractures. Apart from the consistency and reproducibility of a classification system, the simplicity in its clinical application should also be achieved. On one hand, the Neer classification considers the effect of displacement forces exerted on the fracture fragments by their musculotendinous attachments, identifying four main fragments and 16 fracture subtypes. On the other hand, the AO/OTA classification system, based on the Müller classification, considers the progressive severity of the fracture morphology and identifies three main fracture types [23]. Further subgroups are based on the displacement of the fracture fragments, the degree of displacement and the impaction, resulting in a total of 27 fracture subtypes. Although these two systems are very comprehensive and most widely used, their reliability and reproducibility are still controversial [45], [46], [47]. Moreover, both classifications do not allow any statements to be made with regard to prognostic parameters. In 2004, Hertel et al. utilized the already known Codman classification, with an attempt to simplify the practicality by only taking into account displacement of the hinge of >2 mm and the length of the calcar [24]. This allowed a statement to be made about the prognosis with regard to avascular humeral head necrosis. Present comparative studies show that neither the inter- nor the intra-observer reliability of the three different classification systems (Neer, AO/OTA and Hertel) obtained any significant differences [45], [48]. The statement regarding the prognosis of a humerus head fracture, as well as the ease of use, make the Hertel classification a clinically well applicable classification system. Our own data prove that the simplified fracture classification into simple and severe fractures based on the Hertel classification is suitable with regard to the clinical results and the complication rate. Especially in everyday clinical practice, it is of great importance to provide classifications that are easy to assess and help physicians in making therapeutic decisions. Thus, this classification could be an interesting, easily applicable tool. Nonetheless, further studies with higher number of patients and a control group of patients with simple fractures that were treated conservatively will be needed to further support our recent findings. 

A limitation of this study is the fairly high rate of patients that were lost to follow-up (53% of all included patients). We outlined all reasons for exclusion and drop-out in detail (see Figure 1 (Fig. 1)). To our knowledge, this is a general problem of clinical trials in the field of orthopedic trauma due to the heterogeneous patient collective. 

## Conclusion

Our results suggest an early clinical benefit for fixed-angle plate fixation in simple proximal humeral fractures. Although this might only provide a short-term benefit, one could expect an earlier hospital discharge, allowing especially the vulnerable elderly population to carry out their activities of daily life sooner and more independently. However, especially for the elderly, RCTs that compare conservative therapy versus fixed-angle plate fixation in simple proximal humeral fractures are required.

## Notes

### Ethical committee approval

The study was approved by the local institutional review board (IRB) of the Heinrich-Heine-University Düsseldorf. Study number: 5429.

### Competing interests

The authors declare that they have no competing interests.

## Figures and Tables

**Table 1:**
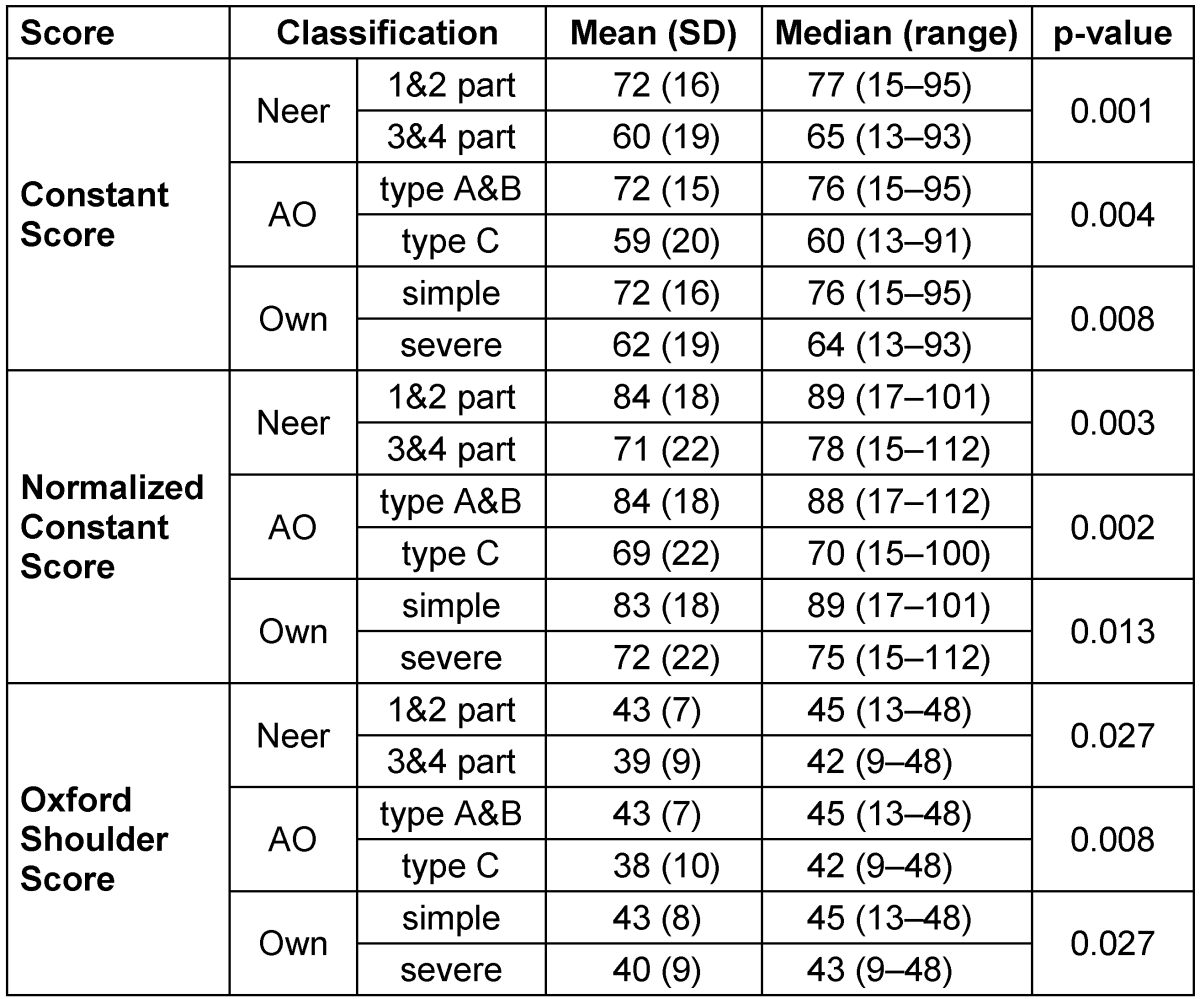
Functional results regarding different fracture classifications Patients with less severe fractures according to the three different classification systems applied achieved significantly better results in each of the three different functional scores.
